# Strigolactones Inhibit Caulonema Elongation and Cell Division in the Moss *Physcomitrella patens*

**DOI:** 10.1371/journal.pone.0099206

**Published:** 2014-06-09

**Authors:** Beate Hoffmann, Hélène Proust, Katia Belcram, Cécile Labrune, François-Didier Boyer, Catherine Rameau, Sandrine Bonhomme

**Affiliations:** 1 Institut Jean-Pierre Bourgin, UMR1318 Institut National de la Recherche Agronomique-AgroParisTech, Versailles, France,; 2 Centre de Recherche de Gif, Institut de Chimie des Substances Naturelles, UPR2301 CNRS, Gif-sur-Yvette, France; Ben-Gurion University of the Negev, Israel

## Abstract

In vascular plants, strigolactones (SLs) are known for their hormonal role and for their role as signal molecules in the rhizosphere. SLs are also produced by the moss *Physcomitrella patens*, in which they act as signaling factors for controlling filament extension and possibly interaction with neighboring individuals. To gain a better understanding of SL action at the cellular level, we investigated the effect of exogenously added molecules (SLs or analogs) in moss growth media. We used the previously characterized *Ppccd8* mutant that is deficient in SL synthesis and showed that SLs affect moss protonema extension by reducing caulonema cell elongation and mainly cell division rate, both in light and dark conditions. Based on this effect, we set up bioassays to examine chemical structure requirements for SL activity in moss. The results suggest that compounds GR24, GR5, and 5-deoxystrigol are active in moss (as in pea), while other analogs that are highly active in the control of pea branching show little activity in moss. Interestingly, the karrikinolide KAR_1_, which shares molecular features with SLs, did not have any effect on filament growth, even though the moss genome contains several genes homologous to *KAI2* (encoding the KAR_1_ receptor) and no canonical homologue to *D14* (encoding the SL receptor). Further studies should investigate whether SL signaling pathways have been conserved during land plant evolution.

## Introduction

Extant bryophytes are considered as descendants of the first plants that colonized land ca 460–470 million years ago. They were able to sustain growth and reproduction in an aerial environment due to their evolutionarily innovative features that could anchor the plant to the soil [1]. *Physcomitrella patens* is a bryophyte from the moss lineage that is now a widespread plant model for studying evolution of plant development mechanisms and diversification of plant architecture [2]. Despite its simple architecture, *P. patens* has developmental mechanisms that are very similar to those of vascular plants, with hormones playing central roles as growth regulators [3].

In *P. patens*, haploid spores germinate and the first cells divide, producing chloronema filaments that very rapidly differentiate into a second type of filament, the caulonema. Chloronema filaments, rich in chloroplasts, only grow in the light. In contrast, caulonema filaments contain fewer chloroplasts, show faster apical cell division and ensure filament extension and colonization of the soil, in the light and in the dark [4]. Chloronema and caulonema filaments both elongate by tip growth [5], and constitute the protonemal network. The subapical cell of caulonema divides asymmetrically to form a bud that goes on to develop a leafy shoot, the gametophore, on which reproductive structures differentiate and fertilization takes place. Rhizoid filaments differentiate from the gametophore and ensure soil anchoring and nutrient uptake.

*P. patens* produces strigolactones (SLs) [6], the most recently discovered plant hormone that inhibits axillary bud outgrowth in vascular plants [7], [8], [9]. SLs are small carotenoid-derived molecules that, as phytohormones, have multiple roles during plant development in addition to inhibiting branching [9], [10]. They regulate root architecture and root hair growth [11], (cambium) secondary growth [12] and plant height [13]. Before the discovery of their phytohormonal properties, SLs were known for their role in the rhizosphere as signals emitted by host plants that promote parasitic plant seed germination [14] and stimulate hyphal proliferation of symbiotic arbuscular mycorrhizal fungi as part of a complex molecular dialogue [15], [16]. It is very likely that SLs are very ancient molecules that played a crucial role in plant adaptation to the terrestrial environment. Recent studies indicate that the primary role of SLs was hormonal: these molecules probably appeared prior to arbuscular mycorrhizal (AM) symbiosis because they have been detected in Charales, which predate the Embryophyta (i.e. land plants) lineage [17].

Mosses possess most genes encoding the key enzymes of SL biosynthesis, namely both carotenoid cleavage dioxygenase (CCD) genes *CCD7* and *CCD8* [6], [18], as well as several homologs of the rice *DWARF27* (*D27*) gene that encodes an isomerase responsible for the first step of trans-β-carotene isomerization [17], [19]. The SL signaling pathway in vascular plants involves the F-box protein MAX2/RMS4/D3, and a member of the α/β hydrolase superfamily, the DWARF14 (D14/DAD2) protein, which is very likely the SL receptor [20], [21], [22], [23]. Very recently, several target proteins have been described, that would be degraded following their recognition by a complex involving MAX2/D3, D14 and an active SL. These include the DWARF53 (D53) rice repressor [24], [25] and the Arabidopsis brassinosteroid transcriptional effector BES1 [26]. SL signaling target proteins and perception factors remain to be described in moss. Differences may concern the SL receptor itself, since only D14-like sequences (and no canonical D14 homolog) have been found in the moss genome[17], [27].

A study of a knock-out mutant for the *CCD8* gene, established that SLs regulate *P. patens* protonema (caulonema and chloronema) branching, and control plant size as quorum-sensing like molecules very likely by controlling caulonema radial extension [6]. However, a better understanding of how SLs inhibit protonema extension in moss is needed, and the cellular effects of SLs have yet to be described, particularly whether SLs inhibit cell division and/or cell elongation. The feedback control on SL synthesis genes, previously characterized in vascular plants [28], has also been highlighted in moss because *PpCCD7* transcripts are upregulated in the SL-deficient *Ppccd8* mutant and SL application decreased *PpCCD7* transcript levels [6].

Exploring the links between the chemical structure of SL molecules and their activity on moss filament cells is useful for determining structural requirements for bioactivity. Comparison of those requirements with regard to hormonal bioactivity in vascular plants and non-vascular plants and with regard to other functions of SL in the rhizosphere may give indications on SL reception in the different systems. To date the SL-receptor has been identified only for the hormonal function in vascular plants [21], [23]. Structure-activity relationship (SAR) studies have already been performed for the main known functions of SLs in vascular plants. Various natural SLs or synthetic analogs have been tested for their activity as a plant hormone (e.g. on pea buds or *Arabidopsis* root hairs) or as a stimulant of parasitic plant seed germination or AM hyphal branching [29], [30], [31], [32], [33]. For all SL functions, the D ring is essential for bioactivity. Although modifications of the tricyclic lactone (ABC ring) have no major effect on pea branching, the ABC ring is essential for AM hyphal branching [31]. The CD part of the molecule is sufficient for activating the SL germination effect on parasitic weeds [30],[34]. In pea, some analogs (e.g. analog **23**, 3′-methyl-GR24) are very active on pea buds but are poorly recognized by parasitic plant seeds, opening the possibility for the use of SLs in agronomy [22], [32], [33]. Natural SLs found in moss and SL analogs with modified ABC rings or D ring with strong bioactivity for the control of shoot branching but not for AM hyphal branching have been tested on moss.

We investigated the cellular effects of SLs on moss in the light and in the dark. Dark-grown moss filaments show negative gravitropism [35]. Since only caulonema filaments grow in dark, caulonema length and caulonema cell sizes can be easily quantified in dark culture conditions. In addition, the use of the SL-deficient *Ppccd8* mutant [6] make it possible to better characterize the effect of exogenous SL added to the growth medium, since this effect is enhanced in comparison with the wild type (WT) which contains endogenous SLs, and as observed in other SAR studies on vascular plants [32], [33], [36], [37]. Here, we show that SLs control filament extension by decreasing the caulonema cell division rate with a slight effect on cell elongation. The moss growth assays conducted after addition of GR24 were very effective, and were used to test the activity of various natural SLs and analogs on filament growth. The effect of karrikins was also tested. These smoke-derived small compounds [38] are butenolides as are SLs, and share certain components of the SL signaling pathway [27]. These assays should further help pinpoint which parts of SL chemical structure are required to inhibit protonema extension in *P. patens* compared to those required for hormonal activity in vascular plants, or for activity in the rhizosphere as part of parasitic and symbiotic relationships [22], [32].

## Results

### Strigolactone effects on moss filaments in the light

**Figure 1:**
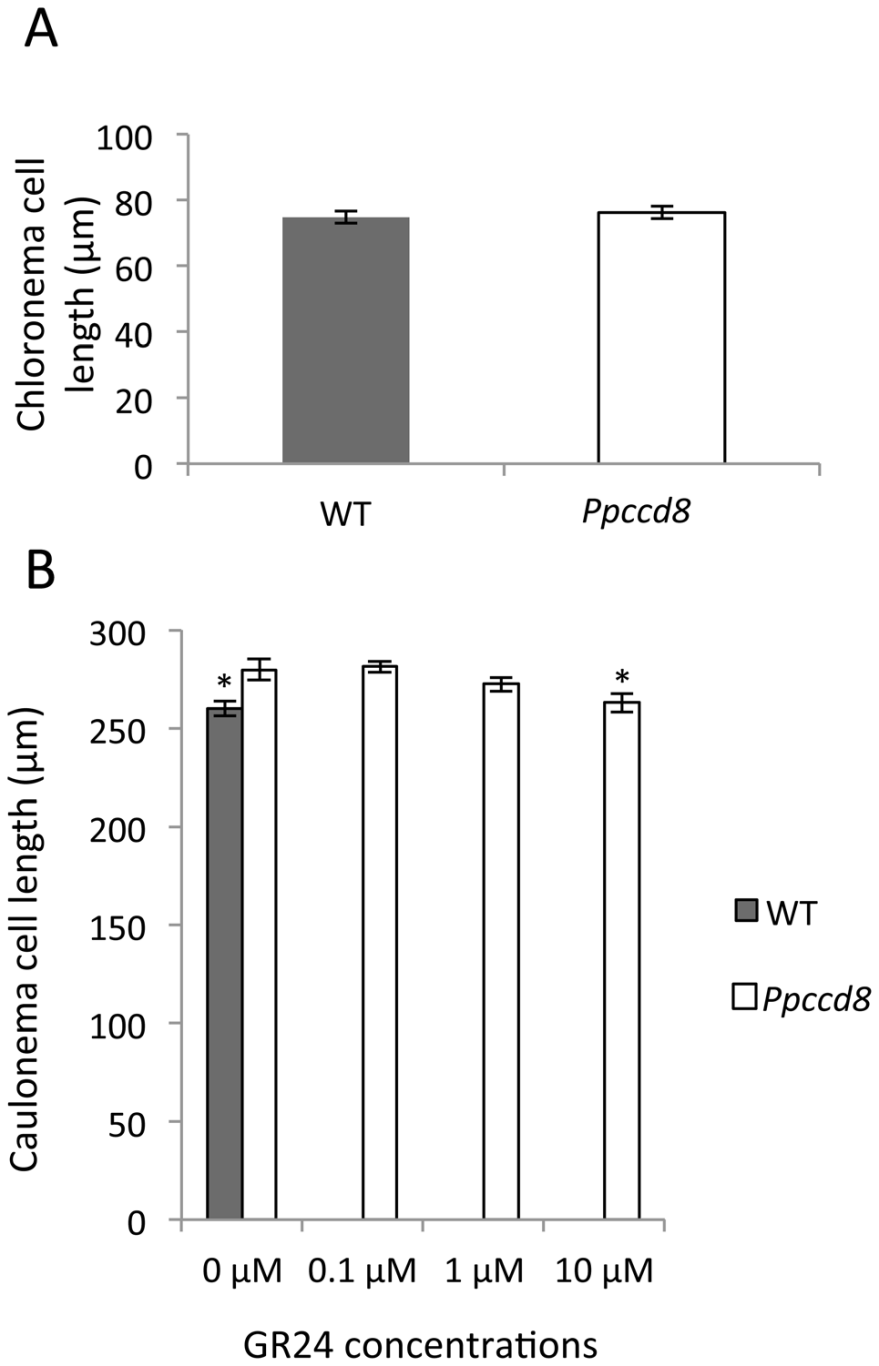
SLs inhibit caulonema cell length but not chloronema cell length in the light. **(A)** Chloronema cell length was measured from protonemata grown for 7 days from fragmented moss. Data are means ± SE (n = 30). **(B)** Caulonema cell length was measured in 26 day-old individuals grown in two Petri dishes. Data are means ± SE (n = 32 to 50 cells). Asterisks denote significant differences from the *Ppccd8* genotype treated with acetone (0 µM GR24) (* *P*<0.05; one-way ANOVA).

To investigate the cellular effects of SLs on *P. patens* protonema in the light, we compared the cell length of chloronema and caulonema filaments from WT and SL-deficient *Ppccd8* mutants in light conditions. Given that the transition from chloronema to caulonema is progressive, chloronema cell length was first quantified on 7-day-old WT and *Ppccd8* protonemata, before the initiation of caulonema cells. This seven-day growth period helped avoid confusion between chloronema cells and the first initiated caulonema cells. There were no statistical differences between WT and *Ppccd8* in chloronema cell length (Figure 1A). In 26-day-old plants, *Ppccd8* caulonema cells were slightly, but significantly, longer than WT caulonema cells (Figure 1B). Addition of GR24 to the *Ppccd8* mutant medium led to a reduction in caulonema cell length (significant only at the highest concentration of GR24, i.e. 10 µM), with a size similar to that of the WT (Figure 1B).

Consequently, no significant difference in cell length was observed for chloronema cells, but significantly longer caulonema cells were observed in the SL-deficient *Ppccd8* mutant. Because the length of light-grown caulonema filaments is not easy to measure, filaments were grown in the dark to test whether SLs also affect cell number, hence cell division.

### Strigolactone effects on moss filaments in the dark

**Figure 2:**
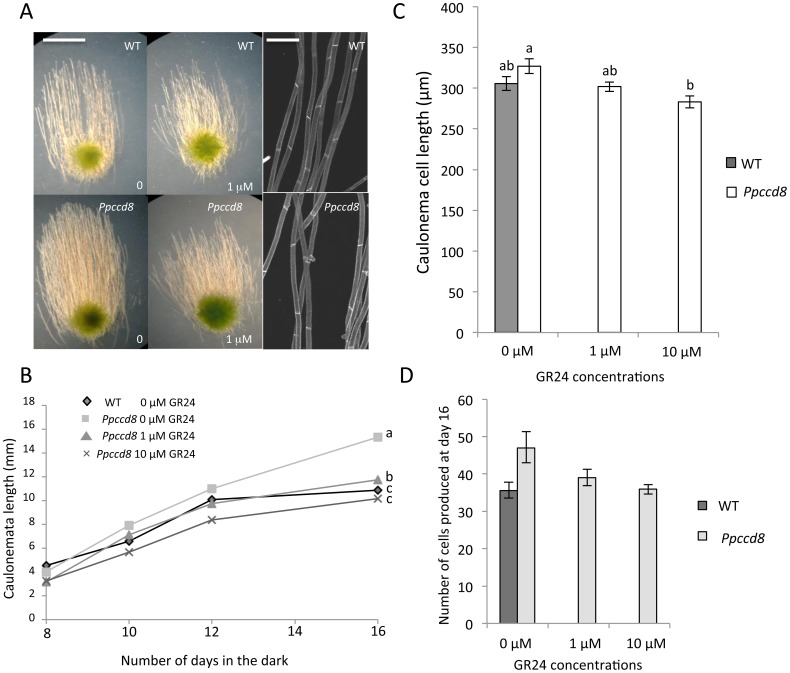
Exogenous strigolactone application reduces growth of caulonemata in the dark by reducing cell length and cell number. **(A)** Left and middle panels: WT (top) and *Ppccd8* mutant (bottom) grown from spores for 16 days in the light, then transferred to the dark for 11 days, without (0) or with GR24 (1 µM). Bar = 5 mm. Right panels: WT (top) and *Ppccd8* mutant (bottom) cells following propidium iodide staining. Bar = 150 µm. **(B)** Caulonema length was quantified after 8 to 16 days in the dark. Data are means ± SE (n = 60–100 filaments from moss fragments grown in two Petri dishes); for day 16, values with the same lowercase letter are not significantly different (one-way ANOVA, *P*<0.01). **(C)** Cell length quantified after 16 days in the dark. Data are means ± SE (n = 50–70 cells). Values with the same lowercase letter are not significantly different (one-way ANOVA, *P*<0.01). **(D)** Number of caulonema cells produced in the dark by day 16 estimated by the ratio of caulonema length to mean cell length at day 16. Minimum and maximum values estimated from confidence intervals of caulonema and cell length (see Methods). The experiment was repeated and confirmed these results (not shown).

In the dark, *P. patens* caulonema filaments grow provided that there is an exogenous source of carbon in the medium. These filaments show negative gravitropism, and entire filaments and single cell lengths can be measured (Figure 2A). Moreover, chloronema filaments do not grow in these conditions, making it easier to observe caulonemata. First, the effects of GR24 on dark-grown caulonema were observed, then other available SL analogues were tested and their effect compared to that of GR24. In addition, since at least 11 *PpD14-like* genes are present in the moss genome [17], [27], belonging to the KAI2 (KAR_1_ receptor [39], [40]) clade, the effect of KAR_1_ was also investigated.

#### Test of GR24 effects on dark-grown caulonemata

To test GR24 effects on dark-grown caulonemata, individuals were first grown in the light for 8 days on SL-free medium, and then transferred to the dark on medium containing fresh GR24. Caulonema length was measured every 2 days from day 8 to day 16 in the dark. After 10 days in the dark, on control plates without GR24, *Ppccd8* caulonemata were longer than the WT caulonemata, indicating that endogenous SLs or derived metabolites also contributed to caulonema length (Figure 2A and 2B). Also note that caulonema filaments were much more numerous in the *Ppccd8* mutant compared to the WT (Figure 2A). After 12 days in the dark, the WT caulonema growth curve reached a plateau. At GR24 concentrations of 1 and 10 µM, *Ppccd8* caulonema size decreased and showed a growth curve similar to that of the WT (Figure 2B). To investigate whether SLs affect cell length and/or cell division, we measured the size of caulonema cells after 16 days in the dark. No significant differences in cell length were observed between the WT and mutant controls (0 µM GR24). As observed for light-grown caulonemata, addition of 1 µM GR24 in the dark had a slight effect on *Ppccd8* mutant cell size, and 10 µM GR24 led to a significant reduction in cell size (Figure 2C). We estimated the number of cells produced by day 16 in the dark by dividing the length of the caulonemata by mean cell length at day 16 (Figure 2D). The minimum and maximum values were calculated from the confidence intervals of caulonema and cell length (see Methods). Within 16 days in the dark, WT plants produced caulonemata with 35.5 cells on average, whereas the *Ppccd8* mutant produced a mean of 46.9 cells. Consequently, the rate of cell division of WT caulonemata in the dark was about 2.2 cells per day, which is close to published values [3], and 2.9 cells per day for *Ppccd8* caulonemata. The addition of 10 µM GR24 to the medium led to a decrease of the caulonema cell division rate, to a value close to that of WT plant (Figure 2D).

Altogether, these results indicate that SLs regulate caulonema cell division, with a limited effect on cell length. These data confirm what was observed on chloronema filament by direct measurements of cell numbers at different times just after spore germination in *Ppccd8* and WT ([6]).

#### Test of various SLs and analogs on caulonema length in the dark

Two types of bioassay were used: the effect on caulonema growth in the dark (filament length at 10 days after adding SLs) and a molecular assay on the expression of the *PpCCD7* gene [6].

**Figure 3:**
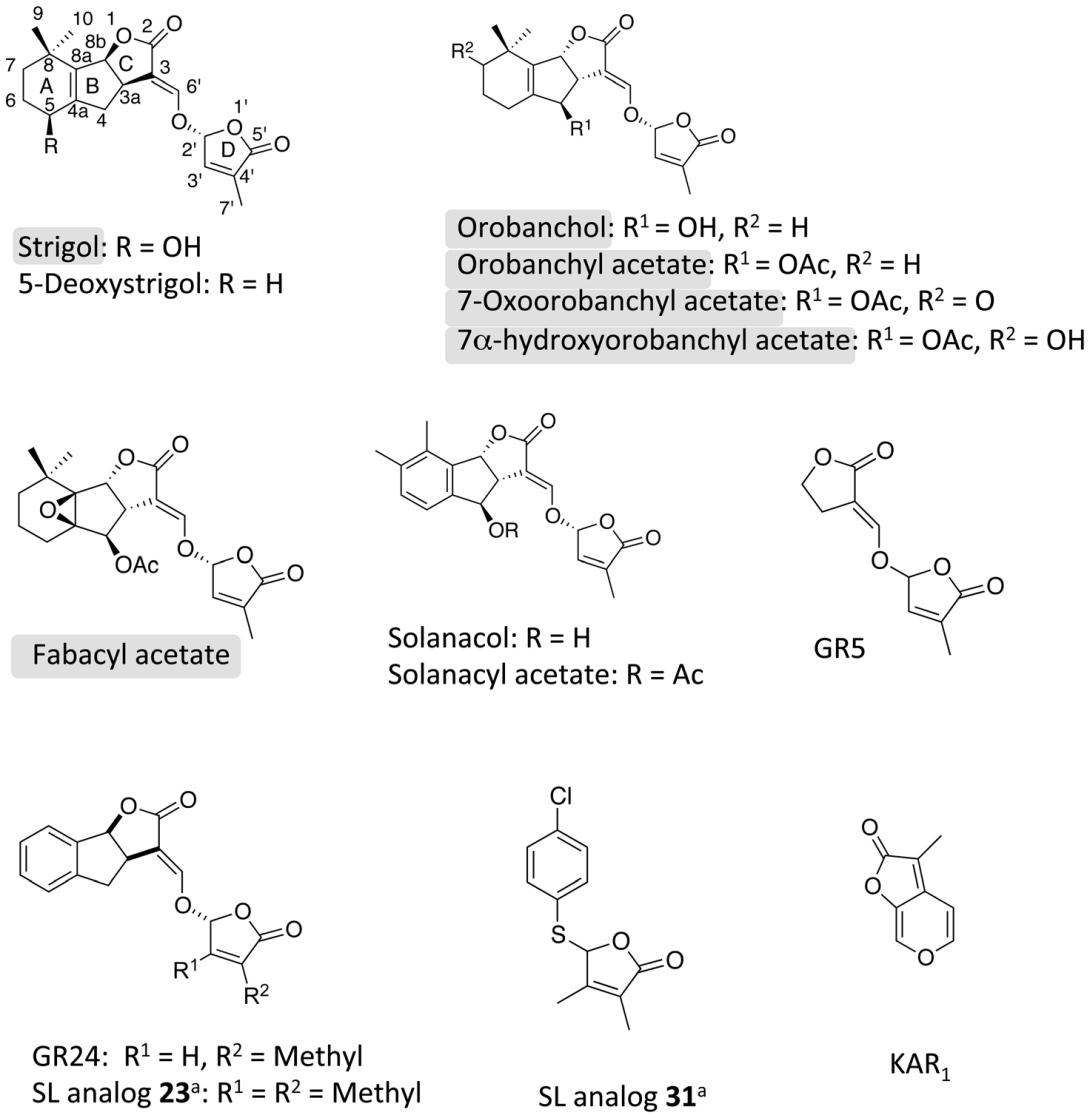
Chemical structures of natural SLs, analogs and KAR_1_. Ac, Acetyl. ^a^ Numbers in bold refer to the numbers assigned to modified SL compounds used for SAR studies in pea (Boyer et al., 2012). Natural strigolactones found in *Physcomitrella* (Proust et al, 2011) are highlighted in gray.

**Figure 4:**
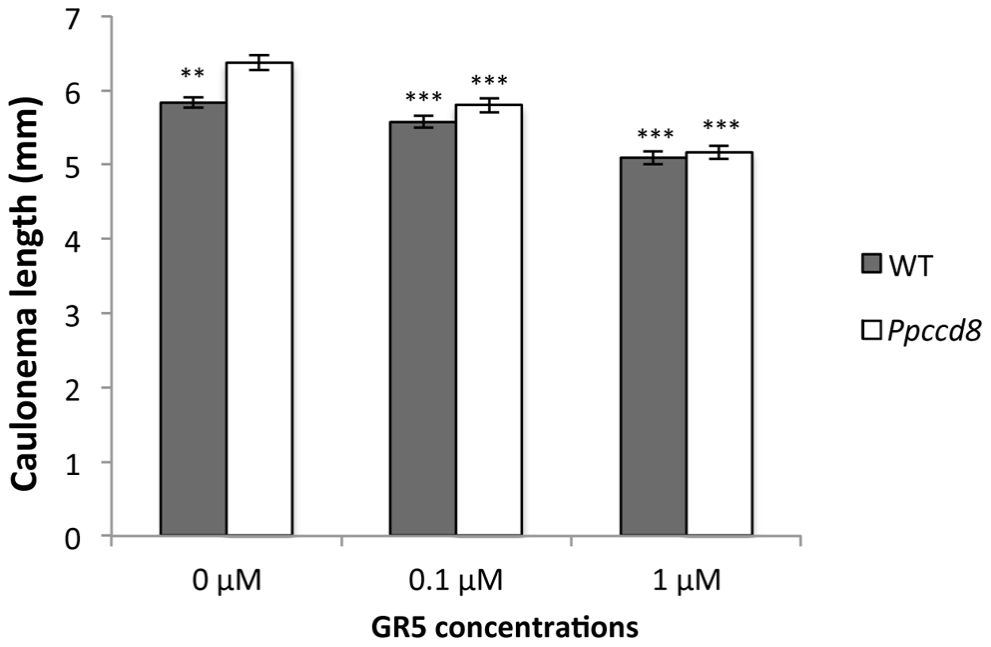
Activity of synthetic SL analog GR5 on dark-grown *P. patens* protonemata. Effect of GR5 on caulonema length after 6 days in the dark. Data are means ± SE (n = 10 to 12 individuals grown in two Petri dishes; ten caulonema per individual). Asterisks denote significant differences from corresponding genotype treated with acetone (0 µM GR5) (*** *P*<0.001, one-way ANOVA).

**Figure 5:**
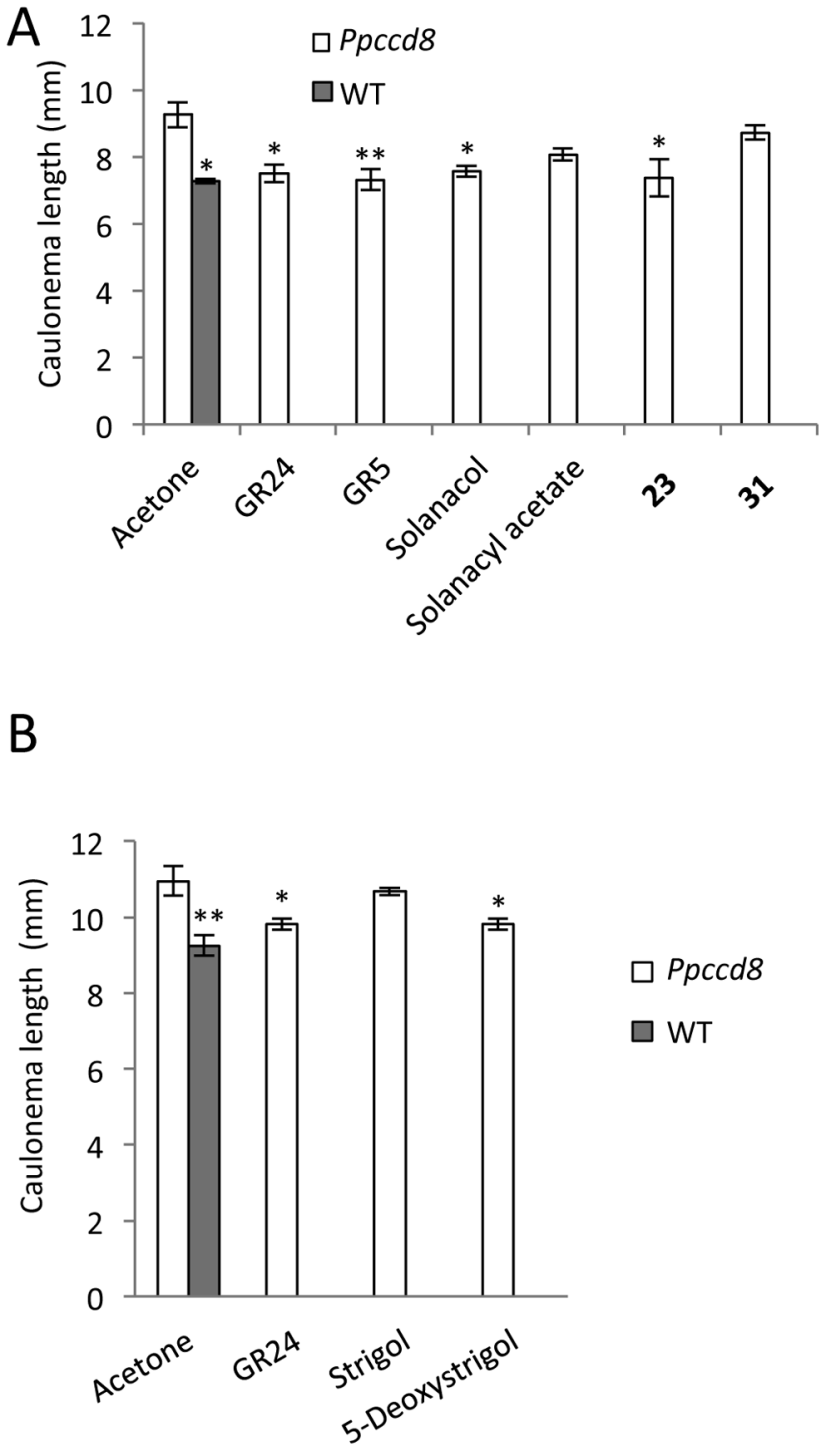
Effect of different natural SLs and analogs (each 1 µM) on caulonema length in the *Ppccd8* mutant after 10 days (A) or 14 days (B) in the dark. Data are means ± SE (n = 2 or 3 Petri dishes for 5A and n = 3 Petri dishes for 5B; 30 to 50 filaments were measured per Petri dish). Asterisks denote significant differences from *Ppccd8* treated with acetone (control) (* *P*<0.05, ** *P*<0.01, one-way ANOVA).

We first tested the activity of a synthetic analog that shows high activity with regard to branching inhibition in pea, and is available in large amount (Figure 3; [32], [33]). Addition of the GR5 analog lacking the A and B rings led to a significant decrease of caulonemata size in *Ppccd8* mutant at 0.1 µM, and in the WT and the *Ppccd8* mutants at 1 µM (Figure 4 *P*<0.001, one-way ANOVA). The same molecule was tested again at 1 µM along with SL analogs GR24, **23** (with two methyl groups on the D ring) and **31** (a thia-3′-methyl-debranone-like molecule) and two natural SLs. All but two tested molecules significantly decreased caulonema length (*P*<0.05, one-way ANOVA) (Figure 5A). The GR5 molecule had effects similar to GR24 on total caulonemata length, as did the natural SL solanacol and the SL analog **23**, which is one of the most effective compounds for inhibition of pea branching [32]. Solanacyl acetate and SL analog **31** showed no significant effects on caulonema growth in the dark. In another similar assay, both strigol and 5-deoxystrigol natural SLs were tested, along with GR24. 5-Deoxystrigol and GR24 had a significant effect on caulonemata length (*P*<0.05, one-way ANOVA), but not strigol (Figure 5B).

#### Do karrikins have an effect on moss caulonemata?

**Figure 6:**
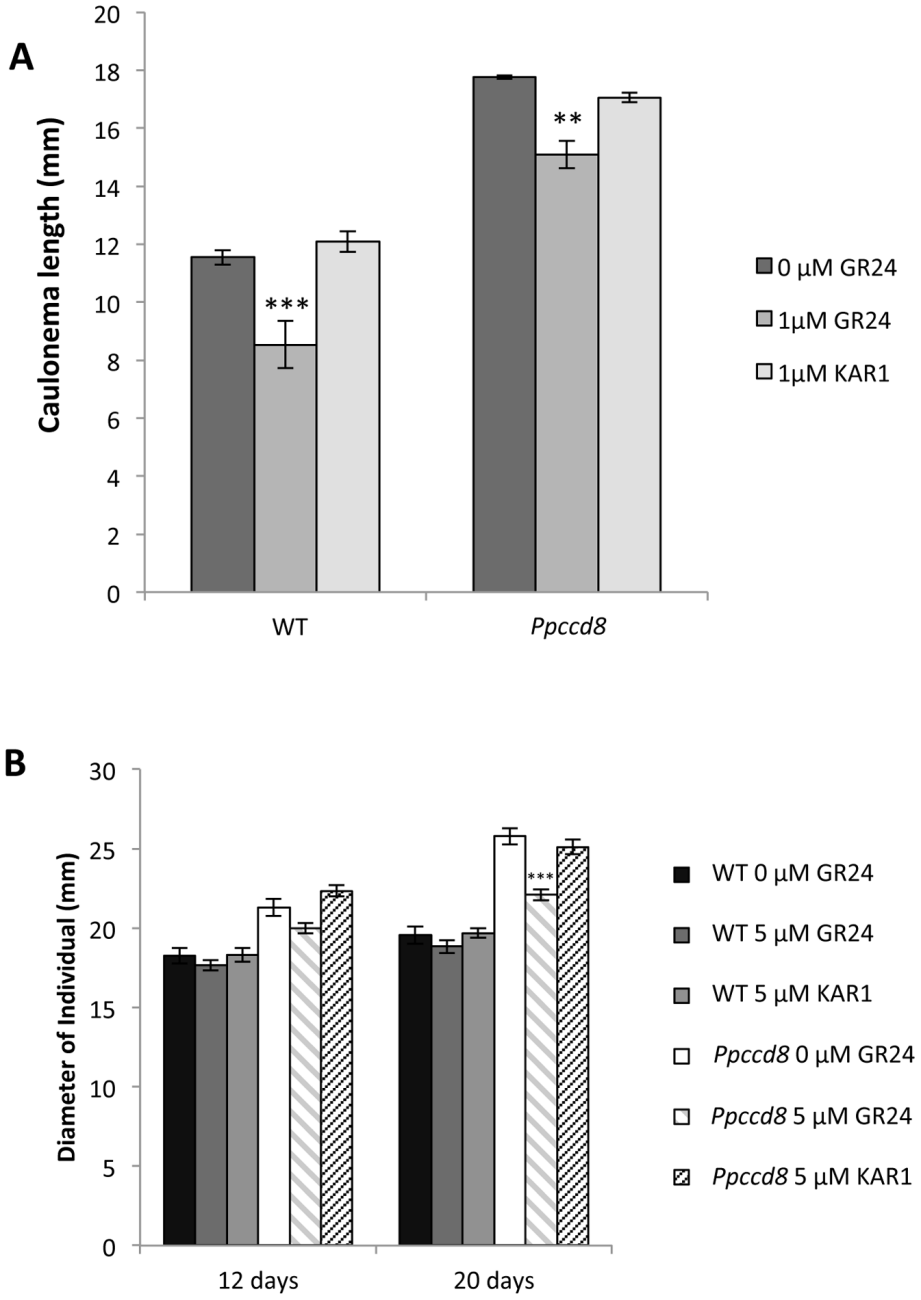
KAR_1_ does not show activity in *P. patens*. **(A)** Effect of GR24 and KAR_1_ (1 µM) on caulonema length after 17 days in the dark. Data are means ± SE (n = 7 to 9 individuals; 10 caulonema per individual). **(B)** Effect of GR24 and karrikinolide (KAR_1_) at 5 µM on the diameter of plants grown in the light after 12 and 20 days of treatment. Data are means ± SE (n = 30 individuals grown from spores on two or three Petri dishes). Asterisks denote significant differences from corresponding genotype treated with acetone (0 µM GR24) (** *P*<0.01, one-way ANOVA, *** *P*<0.001, one-way ANOVA).

As the moss genome contains several genes homologous to *KAI2* (encoding the KAR_1_ receptor) and no canonical homologue to *D14* (encoding the SL receptor, see above), it is tempting to presume an effect of the karrikins on moss development. The activity of KAR_1_ (Figure 3), the first isolated karrikin [38], was tested on moss in the dark and in the light and compared to that of GR24. To do so, 23 day-old individuals grown from spores in the light were transferred to a medium containing KAR_1_ or GR24 at 1 µM, and were placed vertically in the dark. After 17 days in the dark, the length of WT and *Ppccd8* mutant caulonemata showed, compared to controls, a significant decrease in the presence of GR24, but not KAR_1_ (Figure 6A). Because karrikins promote *Arabidopsis* seed germination [41], we tested whether KAR_1_ has an effect on the germination of moss spores. Spores of *P. patens* cannot germinate in the dark and the addition of KAR_1_ to the medium had no effect on spore germination in the dark (data not shown).

Given that KAR_1_ did not have any effect in the dark, a higher concentration of KAR_1_ was used for the light assay (5 µM instead of 1 µM). Eighteen-day-old individuals grown from spores of WT and *Ppccd8* mutant were transferred to media containing KAR_1_ or GR24 and grown on in the light. Plant diameter was measured after 12 and 20 days (Figure 6B). After 12 days, there was a decrease in plant diameter, although not significant, in the presence of GR24, but not KAR_1_. The decrease in mutant diameter was highly significant for the GR24 treatment at 20 days, whereas KAR_1_ still had no effect. The same pattern was observed for WT plants, with a slight decrease, although not significant in plant diameter following the addition of GR24, but not KAR_1_. Hence, the KAR_1_ karrikin does not show any activity on moss caulonema growth in either the light or in the dark. Moreover no particular phenotype was observed on plants grown on KAR_1_ containing medium (data not shown).

#### Molecular assay to test the activity of various SLs in the light

Because these bioassays on moss last several days, the stability of SLs may be an important factor of their activity. Furthermore, SLs are known for their instability in aqueous media [31], [32]. Therefore, we used a molecular assay to measure the levels of *PpCCD7* transcripts in plants after the addition of SL. We have previously shown [6] that treatment with GR24 downregulates *PpCCD7* gene expression in the *Ppccd8* mutant, and thus restores the feedback control on SL synthesis genes observed in the WT.

**Figure 7:**
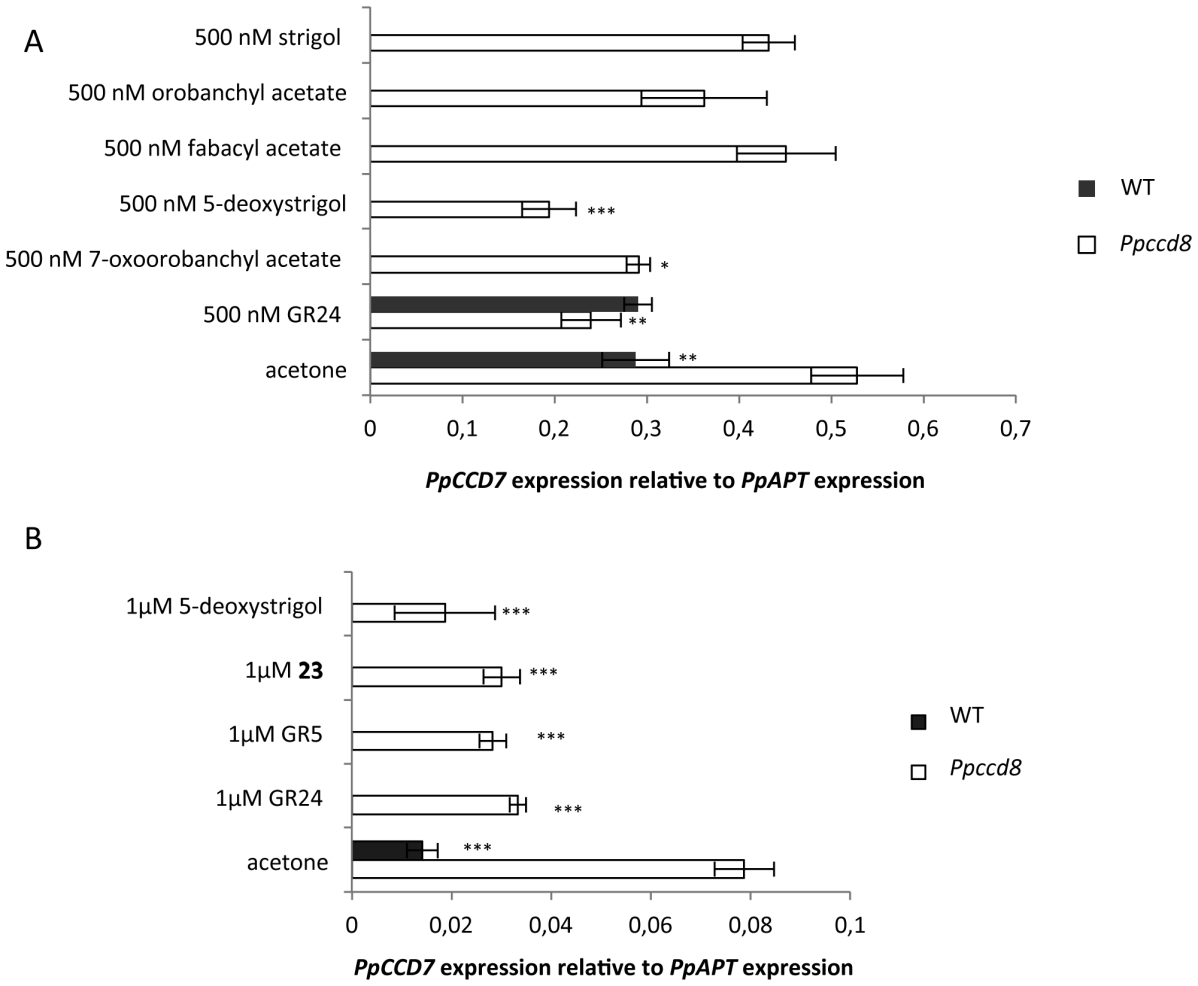
Effect of different strigolactones and analogues on the relative *PpCCD7* expression in the light. **A**: Transcript level 24 h after application of 500 nM SL. Data are means ± SE (n = 3 or 4 biological replicates). Asterisks denote significant differences from *Ppccd8* treated with acetone (* *P*<0.05, ** *P*<0.01, *** *P*<0.001, one-way ANOVA). **B**: Transcript level 6 h after application of 1 µM SL. Data are means ± SE (n = 3 biological replicates). Asterisks denote significant differences from *Ppccd8* treated with acetone (*** *P*<0.001, one-way ANOVA).

In the first experiment, the levels of *PpCCD7* transcripts were measured 24 h after the addition of SLs (at 500 nM). The lack of feedback control in the *Ppccd8* mutant led to a relative *PpCCD7* transcript level almost twice as high as that in the WT (Figure 7A). Addition of GR24 or 5-deoxystrigol at 500 nM to the mutant culture led to a significantly lower level of *PpCCD7* transcripts (*P*<0.01, one-way ANOVA), which was comparable to that of WT. Addition of 7-oxoorobanchyl acetate also showed a significant effect on *PpCCD7* transcript levels (*P*<0.05, one-way ANOVA). However, fabacyl acetate, orobanchyl acetate and strigol, all of which are natural SLs found in moss exudates [6], showed no significant effect at 500 nM (Figure 7A). In the second experiment, synthetic analogs GR5, GR24 and **23**, together with 5-deoxystrigol (natural SL) were added at 1 µM to the medium, and the *PpCCD7* transcript level was measured 6 h after application (Figure 7B). In this experiment, the *PpCCD7* transcript level in the *Ppccd8* mutant was more than three times higher than in the WT (Figure 7B). All four SLs or SL analogs led to a significant decrease in *PpCCD7* transcript levels in the *Ppccd8* mutant (*P*<0.001, one-way ANOVA). In the third experiment, the activity of three natural molecules (strigol, fabacyl acetate and 5-deoxystrigol) was tested as early as 2 h following application of 100 nM SL (Figure S1). In these conditions, only GR24 showed a significant effect on *PpCCD7* transcript levels (*P*<0.05, one-way ANOVA), although the addition of 5-deoxystrigol also seemed to decrease *PpCCD7* expression compared to the acetone-treated control.

#### Molecular assay in the dark

Since the effect of SLs on caulonema elongation was observed on dark-grown caulonemata, we also measured *PpCCD7* transcript level after transfer to SL-containing medium in the dark. Similar to results in light-grown caulonemata, the feedback control on *PpCCD7* transcript levels was observed in the *Ppccd8* mutant 6 h after the addition of 1 µM GR24 in the dark (Figure S2). Moreover, *PpCCD7* transcript levels also decreased following transfer to medium in which WT plants had grown and produced natural SLs. As observed in light-grown plants, transfer to media on which *Ppccd8* plants had grown had no effect on *Ppccd8* mutant *PpCCD7* transcript levels in the dark (Figure S2). To conclude, natural SL exudates from moss appear to be as effective as GR24 in restoring the feedback control on *PpCCD7* transcript levels in the dark. These results corroborate the effect of SLs on the dark-grown caulonema phenotype.

## Discussion

### SLs inhibit caulonema elongation

When grown in the light, WT *P. patens* plants stop growing after 3 weeks, whereas the SL-deficient *Ppccd8* mutant does not stop growing. Transfer of the mutant onto medium containing the synthetic SL GR24 restores plant diameter to that of the WT [6]. Here, we showed that exogenous application of the synthetic SL GR24 inhibits mutant growth by controlling caulonemata growth in the light and in the dark. Dark-grown moss is more convenient for characterizing caulonema filaments and cells, independently of chloronema. In the dark, 1 µM GR24 was needed to restore the *Ppccd8* mutant phenotype to a WT phenotype, and only the highest concentration (10 µM GR24) had a significant effect on the length of caulonema cells, suggesting that GR24 has a stronger effect on cell division than on cell length (see below).

Contrary to what is observed on *P. patens* caulonemata, addition of GR24 promotes root-hair elongation in the vascular plant *Arabidopsis* [42]. Root hairs, like caulonema cells, elongate by tip growth due to active exocytosis at the apical end of the cell [5], [43]. Root hairs and caulonema cells however do not share the same function, and the moss cells most comparable to root hairs are rhizoids, which are involved in water uptake and attachment to the soil. Interestingly, rhizoids are longer in WT *P. patens* than in *Ppccd8* mutants, and the addition of GR24 (10 nM) restores the mutant phenotype, and also positively affects WT rhizoid length [17]. Further work needs to be done to determine whether SLs affect moss rhizoid cell number and/or length.

In moss, caulonemata are also involved in soil/medium exploration, a function that is fulfilled by roots in vascular plants. In *Arabidopsis*, lateral root formation is enhanced in SL synthesis mutants, and to a greater extent in the SL perception mutant *max2* [42], [44]. Addition of GR24 leads to fewer secondary roots in *Arabidopsis* SL synthesis mutants. However, SL effects on lateral root density depends on the nutrient (phosphate, Pi) status of the plant, and contradictory observations have been reported under Pi-limited conditions [45]. In our growth conditions, Pi levels are probably sufficient (2 mM KH_2_PO_4_), and SLs inhibited caulonema growth and density, similar to the inhibitory effect observed on lateral roots in vascular plants. Hence, in moss as in vascular plants, SLs may enhance and inhibit organ size and number, depending upon the organ (rhizoid or caulonema).

### SLs inhibit cell division rather than cell elongation in moss

Our results on moss indicate that, at the cellular level, the caulonema cell division rate is inhibited by exogenously supplied SLs, in contrast to cell length that is only slightly affected. The higher branching rate of protonemata observed in the *Ppccd8* mutant compared to the WT [6] can be attributed to this higher rate of division. More divisions may result in a higher number of caulonemata in the *Ppccd8* mutant. This effect of SLs on cell division rather than cell elongation was already suggested by the higher number of cells per chloronema filament at different times after germination, in *Ppccd8* in comparison to the WT[6].

In vascular plants, SLs have been reported to inhibit or enhance cell division, depending on the species and the tissue in question. In rice, on WT and SL synthesis mutant plants grown in the dark, addition of GR24 negatively regulates mesocotyl cell division but has no effect on cell elongation [46]. In contrast, in *Arabidopsis*, SL synthesis mutants show reduced cambium activity and local treatment of stems with GR24 induces cambium-like cell proliferation [12]. Also in *Arabidopsis* and rice, both SL synthesis and perception mutants show a fewer root meristem cells compared to the WT [44], [47]. In *Arabidopsis*, the addition of GR24 in the growth medium restores the number of root meristem cells in SL-deficient plants [44]. In pea, SL-deficient mutants are relatively dwarf, and this dwarfism is not due to more frequent branching, but to a deficiency in SLs. Dwarfism of the SL synthesis mutant is maintained even when branching is inhibited, and adding GR24 restores internode length [13]. Internodes in dwarf mutants show fewer epidermal cells whose length is not affected, suggesting that SLs stimulate internode elongation by stimulating cell division [13]. SLs were first identified as phytohormones that inhibit axillary bud outgrowth [7], [8]. This hormonal action further suggests that in vascular plants, SLs can have different roles on cell division in different types of meristem. It has been suggested that SLs function as central modulators in plant architecture regulation, allowing the plant to respond to changing environmental (e.g. light) conditions [9], [12].

### Comparison of SARs for SLs between pea (branching inhibition) and moss

We developed two relatively simple bioassays to test the activity of SL analogs in *P. patens*: one based on caulonema growth in dark conditions and one using the feedback control of SL on transcript levels of the biosynthesis gene *PpCCD7*. This feedback control is also observed in the dark, using synthetic GR24 or natural strigolactones (or their derivatives) exuded by the WT moss. Using these bioassays, the bioactivity of five natural SLs and four synthetic analogs were tested in moss to compare with SAR studies performed in pea with regard to branching inhibition [32] and with regard to SL functions in the rhizosphere [30], [31], [48].

In pea, acetate-SLs are always more active than their corresponding hydroxyl-SLs. Fabacyl-acetate, orobanchyl acetate or solanacyl acetate are particularly active, occasionally even at a concentration of 10 nM [32]. Although, strictly speaking, these SLs cannot be directly compared here, all acetate-SLs tested in our bioassay except 7-oxoorobanchyl acetate, showed no significant activity. Similar to what is observed in pea, 5-deoxystrigol was more active than strigol in moss. It is surprising that among the tested molecules, except 7-oxoorobanchyl acetate, those previously detected in moss exudates (i.e. fabacyl acetate, orobanchyl acetate and strigol [6] were not the most active. Similarly, in pea, strong differences of bioactivity have been observed among endogenous SLs as regard to the control of shoot branching[32]. As transfer onto medium in which WT plants had grown had an effect in the molecular assay, this could suggest that active natural SLs are different in moss. 5-deoxy strigol was the most active SL in our phenotypic and molecular bioassays. 5-deoxystrigol is also among the most active compounds in AM fungi [31]. Although all the steps in the SL synthesis pathway have not yet been fully described, 5-deoxystrigol and its isomers are assumed to be the first SLs from which all others are synthesized [30].

The CD-rings analog GR5, which shows a similar level of activity as GR24 in pea, also showed activity comparable to GR24 in *P. patens*. Therefore, ABC rings are not required for SL activity in moss, in contrast to SL structural requirements for AM fungi [31], [33]. The two analogs showing the highest activity in pea, **23** and **31** [32], showed less activity and no significant activity, respectively, compared to GR24 in our phenotypic and molecular bioassays. In rice, a molecule similar to **31** has been shown to be highly active [36]; therefore there may be differences between vascular and non-vascular plants in SL signaling. The *MAX1* gene encoding a cytochrome P450 enzyme involved in the last steps of SL synthesis in vascular plants [49] is absent from the genome of *P. patens* [6], suggesting that another P450 may ensure the same function in moss, or that the SL synthesis pathway is slightly different [22]. Further studies are needed to test the effect of the SL precursor carlactone [19], and its derivatives in mosses, and further determine the differences in SL pathways between vascular and non-vascular plants.

### SL and KAR perception in moss

The karrikinolide KAR_1_ and the synthetic SL GR24 both promote seed germination and inhibit hypocotyl elongation in *Arabidopsis*, and the MAX2 F-box protein is needed for these effects during *Arabidopsis* seedling development [50]. SLs regulate shoot branching via MAX2 and AtDWARF14 (AtD14), whereas KAR_1_ needs MAX2 and AtD14-like/KARRIKIN INSENSITIVE 2 (KAI2) [27]. No effect of KAR_1_ was observed in our bioassay based on caulonema growth in the light and in the dark, despite the fact that there is one homolog of *MAX2* as well as several homologs of *AtD14-like/KAI2* genes in the genome of *P. patens* [27], [51]. In *P. patens*, the function of these genes is still unknown, in particular whether the *PpMAX2* gene is indeed involved in the SL signaling pathway, together with one of the moss KAI2 homologs.

Because SLs in moss inhibit caulonema growth, to distinguish between SL activity and toxicity, a *P. patens* SL response mutant is needed. This kind of mutant is expected to show a phenotype similar to that of the *Ppccd8* SL synthesis mutant, but the phenotype should not be affected by the addition of GR24. A response mutant would also allow the cloning of the gene(s) encoding the receptor(s) and thus represent an important step towards a better understanding of the SL signaling pathway in moss. SLs have been shown to stimulate rhizoid elongation in *P. patens* [17] and another bioassay should be designed to test and to quantify the activity of SL analogs in moss rhizoids. The recent development of fluorescent (or labeled) SL analogs [37], [52] may offer efficient tools for this task, particularly for localizing SLs and their derivatives in plant tissues and comparing SL signaling in vascular and non-vascular plants.

## Methods

### Plant growth conditions

The Gransden WT strain [53] was used along with the *Ppccd8* mutant [6]. Moss protonema were grown on PP-NO_3_ medium [54] for phenotypic observation, and on PP-NO_3_ medium supplemented with 2.7 mM NH_4_-tartrate for propagation. Plants were cultivated either in 9 cm round (for the light experiments) or 12 cm square (for the dark experiments) Petri dishes, on medium solidified with 0.7% agar (Vitro Agar, KALYS SA, France) and overlaid with cellophane (Cannings, Bristol, UK). For dark conditions, 0.5% glucose was added to the medium, 1% Phytoblend agar (Caisson, USA) was used, and Petri dishes were positioned vertically for better observation of caulonema growth. For the light experiments, cultures were placed in growth chambers set at 60% humidity, and with 16 h of light (quantum irradiance of 80 µE m^−2^ s^−1^) at 24°C and 8 hours of dark at 22°C.

### Plant treatments and measurements

Moss plants were either grown from spores, directly plated out on cellophane and grown for 2 weeks in light, or grown from fragmented protonema, using 7-day-old cultures. For dark-grown cultures, a horizontal band of fragmented moss was deposited on the cellophane and first grown in the light. After 8–10 days, each cellophane was transferred to square Petri dishes filled with PP-NO_3_ medium [54] and supplemented with 0.5% glucose, with or without SLs according to treatment, and placed in the dark for an additional 8–18 days. SLs and SL analogs were supplied as described in [6], [32] and [55]. SLs and SL analogs used in the experiments, are racemates. KAR_1_ was generously supplied by Gavin Flematti (University of Western Australia, Crawley Campus). SLs, SL analogs and KAR_1_ were dissolved in acetone and added to the liquid medium cooled to 45°C. Control treatments consisted in adding an equivalent amount of acetone. The Petri dishes were examined under a Leica MZ6 stereo microscope and images of caulonemata were captured by a Nikon CoolPix 4500 camera. Cell images were either captured directly under the microscope (Leitz), or following staining of the filaments with propidium iodide (10 µg/mL for 10 min) prior to transferring them to water on a slide for better visibility of the cross walls of the cells. Imaging of propidium iodide-stained tissue was performed with a Zeiss LSM 710 confocal microscope. The excitation wavelengths were 488 and 561 nm, and emission was collected at 565 to 720 nm. Chloroplasts were also visible, clearly distinguishing the chloronema from the caulonema cells. Measurements were performed with ImageJ software (http://rsbweb.nih.gov/ij/).

### Gene expression analysis

*PpCCD7* gene expression was quantified using real-time PCR as described in [6]. *PpAPT*, which encodes ADENOSINE PHOSPHORIBOSYL TRANSFERASE was used as the constitutive gene.

### Statistical analysis

Tukey's multiple comparison ANOVAs were generally performed for statistical analyses using R Commander version 1.7–3 [56].

The mean number of caulonema cells produced in the dark experiments was estimated by the ratio of the mean caulonema length to the mean of cell length. To estimate the range of possible variation in the number of cells, the minimum and maximum values were estimated from the 95% confidence intervals of caulonema length (LCAU) and caulonema cell length (LCELL). Supposing that these confidence intervals range from LCAU_inf_ to LCAU_sup_ for caulonema length and LCELL_inf_ to LCELL_sup_ for cell length, the minimum value for cell number was estimated by the ratio of LCAU_inf_ to LCELL_sup_ and the maximum value by the ratio of LCAU_sup_ to LCELL_inf_.

## Supporting Information

Figure S1 **Effect of various strigolactones and analogues on relative** ***PpCCD7*** **expression 2 h after application of 100 nM SL in WT and SL-deficient (** ***Ppccd8*** **) mutant plants in the light.** Data are means ± SE (n = 3 biological replicates). Asterisks denote significant differences from *Ppccd8* treated with acetone (* *P*<0.05, one-way ANOVA).
(TIF)

Figure S2 **Relative** ***PpCCD7*** **expression of** ***Ppccd8*** **mutant grown in the light (top) or in the dark (bottom), 6 h after addition of 1 µM GR24 (control: acetone) or following transfer to medium on which WT or** ***Ppccd8*** **mutant plants (fragmented protonema using 7-day-old culture) had grown for 20 days.** Plants from three Petri dishes were used for each condition. A biological replication of the experiment gave similar results.
(TIF)

## References

[1] Ligrone R, Duckett JG, Renzaglia KS (2012) Major transitions in the evolution of early land plants: a bryological perspective. Annals of botany 109: 851–871. 22356739 10.1093/aob/mcs017 PMC3310499

[2] Schaefer DG, Zryd JP (2001) The moss Physcomitrella patens, now and then. Plant physiology 127: 1430–1438. 11743086 PMC1540175

[3] Bonhomme S, Nogue F, Rameau C, Schaefer DG (2013) Usefulness of Physcomitrella patens for studying plant organogenesis. Methods in molecular biology 959: 21–43. 23299666 10.1007/978-1-62703-221-6_2

[4] Cove D, Bezanilla M, Harries P, Quatrano R (2006) Mosses as model systems for the study of metabolism and development. Annu Rev Plant Biol 57: 497–520. 16669772 10.1146/annurev.arplant.57.032905.105338

[5] Menand B, Calder G, Dolan L (2007) Both chloronemal and caulonemal cells expand by tip growth in the moss Physcomitrella patens. Journal of experimental botany 58: 1843–1849. 17404383 10.1093/jxb/erm047

[6] Proust H, Hoffmann B, Xie X, Yoneyama K, Schaefer DG, et al (2011) Strigolactones regulate protonema branching and act as a quorum sensing-like signal in the moss Physcomitrella patens. Development 138: 1531–1539. 21367820 10.1242/dev.058495

[7] Gomez-Roldan V, Fermas S, Brewer PB, Puech-Pages V, Dun EA, et al (2008) Strigolactone inhibition of shoot branching. Nature 455: 189–194. 18690209 10.1038/nature07271

[8] Umehara M, Hanada A, Yoshida S, Akiyama K, Arite T, et al (2008) Inhibition of shoot branching by new terpenoid plant hormones. Nature 455: 195–200. 18690207 10.1038/nature07272

[9] Brewer PB, Koltai H, Beveridge CA (2013) Diverse roles of strigolactones in plant development. Molecular plant 6: 18–28. 23155045 10.1093/mp/sss130

[10] Xie X, Yoneyama K (2010) The strigolactone story. Annu Rev Phytopathol 48: 93–117. 20687831 10.1146/annurev-phyto-073009-114453

[11] Koltai H (2011) Strigolactones are regulators of root development. The New phytologist 190: 545–549. 21638793 10.1111/j.1469-8137.2011.03678.x

[12] Agusti J, Herold S, Schwarz M, Sanchez P, Ljung K, et al (2011) Strigolactone signaling is required for auxin-dependent stimulation of secondary growth in plants. Proceedings of the National Academy of Sciences of the United States of America 108: 20242–20247. 22123958 10.1073/pnas.1111902108 PMC3250165

[13] de Saint Germain A, Ligerot Y, Dun EA, Pillot JP, Ross JJ, et al (2013) Strigolactones Stimulate Internode Elongation Independently of Gibberellins. Plant physiology 163: 1012–1025. 23943865 10.1104/pp.113.220541 PMC3793021

[14] Cook CE, Whichard LP, Turner B, Wall ME, Egley GH (1966) Germination of Witchweed (Striga lutea Lour.): Isolation and Properties of a Potent Stimulant. Science 154: 1189–1190. 17780042 10.1126/science.154.3753.1189

[15] Akiyama K, Matsuzaki K, Hayashi H (2005) Plant sesquiterpenes induce hyphal branching in arbuscular mycorrhizal fungi. Nature 435: 824–827. 15944706 10.1038/nature03608

[16] Nadal M, Paszkowski U (2013) Polyphony in the rhizosphere: presymbiotic communication in arbuscular mycorrhizal symbiosis. Current opinion in plant biology 16: 473–479. 23834765 10.1016/j.pbi.2013.06.005

[17] Delaux PM, Xie X, Timme RE, Puech-Pages V, Dunand C, et al (2012) Origin of strigolactones in the green lineage. The New phytologist 195: 857–871. 22738134 10.1111/j.1469-8137.2012.04209.x

[18] Rensing SA, Lang D, Zimmer AD, Terry A, Salamov A, et al (2008) The Physcomitrella genome reveals evolutionary insights into the conquest of land by plants. Science 319: 64–69. 18079367 10.1126/science.1150646

[19] Alder A, Jamil M, Marzorati M, Bruno M, Vermathen M, et al (2012) The path from beta-carotene to carlactone, a strigolactone-like plant hormone. Science 335: 1348–1351. 22422982 10.1126/science.1218094

[20] Beveridge CA, Kyozuka J (2010) New genes in the strigolactone-related shoot branching pathway. Curr Opin Plant Biol 13: 34–39. 19913454 10.1016/j.pbi.2009.10.003

[21] Hamiaux C, Drummond RS, Janssen BJ, Ledger SE, Cooney JM, et al (2012) DAD2 is an alpha/beta hydrolase likely to be involved in the perception of the plant branching hormone, strigolactone. Current biology: CB 22: 2032–2036. 22959345 10.1016/j.cub.2012.08.007

[22] de Saint Germain A, Bonhomme S, Boyer FD, Rameau C (2013) Novel insights into strigolactone distribution and signalling. Current opinion in plant biology 16: 583–589. 23830996 10.1016/j.pbi.2013.06.007

[23] Nakamura H, Xue YL, Miyakawa T, Hou F, Qin HM, et al (2013) Molecular mechanism of strigolactone perception by DWARF14. Nature communications 4: 2613. 10.1038/ncomms3613 24131983

[24] Zhou F, Lin Q, Zhu L, Ren Y, Zhou K, et al (2013) D14-SCF(D3)-dependent degradation of D53 regulates strigolactone signalling. Nature 504: 406–410. 24336215 10.1038/nature12878 PMC4096652

[25] Jiang L, Liu X, Xiong G, Liu H, Chen F, et al (2013) DWARF 53 acts as a repressor of strigolactone signalling in rice. Nature 504: 401–405. 24336200 10.1038/nature12870 PMC5802366

[26] Wang Y, Sun S, Zhu W, Jia K, Yang H, et al (2013) Strigolactone/MAX2-induced degradation of brassinosteroid transcriptional effector BES1 regulates shoot branching. Dev Cell 27: 681–688. 24369836 10.1016/j.devcel.2013.11.010

[27] Waters MT, Nelson DC, Scaffidi A, Flematti GR, Sun YK, et al (2012) Specialisation within the DWARF14 protein family confers distinct responses to karrikins and strigolactones in Arabidopsis. Development 139: 1285–1295. 22357928 10.1242/dev.074567

[28] Dun EA, Brewer PB, Beveridge CA (2009) Strigolactones: discovery of the elusive shoot branching hormone. Trends in plant science 14: 364–372. 19540149 10.1016/j.tplants.2009.04.003

[29] Cohen M, Prandi C, Occhiato EG, Tabasso S, Wininger S, et al (2013) Structure-function relations of strigolactone analogs: activity as plant hormones and plant interactions. Molecular plant 6: 141–152. 23220943 10.1093/mp/sss134

[30] Zwanenburg B, Pospisil T (2013) Structure and activity of strigolactones: new plant hormones with a rich future. Molecular plant 6: 38–62. 23204499 10.1093/mp/sss141

[31] Akiyama K, Ogasawara S, Ito S, Hayashi H (2010) Structural requirements of strigolactones for hyphal branching in AM fungi. Plant & cell physiology 51: 1104–1117. 20418334 10.1093/pcp/pcq058 PMC2900820

[32] Boyer FD, de Saint Germain A, Pillot JP, Pouvreau JB, Chen VX, et al (2012) Structure-activity relationship studies of strigolactone-related molecules for branching inhibition in garden pea: molecule design for shoot branching. Plant physiology 159: 1524–1544. 22723084 10.1104/pp.112.195826 PMC3428777

[33] Boyer FD, de Saint Germain A, Pouvreau JB, Clave G, Pillot JP, et al.. (2013) New Strigolactone Analogues as Plant Hormones with Low Activities in the Rhizosphere. Molecular plant. 10.1093/mp/sst163 24249726

[34] Zwanenburg B, Mwakaboko AS, Reizelman A, Anilkumar G, Sethumadhavan D (2009) Structure and function of natural and synthetic signalling molecules in parasitic weed germination. Pest Manag Sci 65: 478–491. 19222046 10.1002/ps.1706

[35] Jenkins GI, Courtice GR, Cove DJ (1986) Gravitropic responses of wild-type and mutant strains of the moss Physcomitrella patens. Plant, cell & environment 9: 637–644. 10.1111/j.1365-3040.1986.tb01621.x 11540950

[36] Fukui K, Ito S, Ueno K, Yamaguchi S, Kyozuka J, et al (2011) New branching inhibitors and their potential as strigolactone mimics in rice. Bioorg Med Chem Lett 21: 4905–4908. 21741836 10.1016/j.bmcl.2011.06.019

[37] Rasmussen A, Heugebaert T, Matthys C, Van Deun R, Boyer FD, et al (2013) A fluorescent alternative to the synthetic strigolactone GR24. Molecular plant 6: 100–112. 23024210 10.1093/mp/sss110

[38] Flematti GR, Ghisalberti EL, Dixon KW, Trengove RD (2004) A compound from smoke that promotes seed germination. Science 305: 977. 15247439 10.1126/science.1099944

[39] Guo Y, Zheng Z, La Clair JJ, Chory J, Noel JP (2013) Smoke-derived karrikin perception by the alpha/beta-hydrolase KAI2 from Arabidopsis. Proceedings of the National Academy of Sciences of the United States of America 110: 8284–8289. 23613584 10.1073/pnas.1306265110 PMC3657771

[40] Waters MT, Smith SM (2013) KAI2- and MAX2-mediated responses to karrikins and strigolactones are largely independent of HY5 in Arabidopsis seedlings. Molecular plant 6: 63–75. 23142794 10.1093/mp/sss127

[41] Nelson DC, Riseborough JA, Flematti GR, Stevens J, Ghisalberti EL, et al (2009) Karrikins discovered in smoke trigger Arabidopsis seed germination by a mechanism requiring gibberellic acid synthesis and light. Plant Physiol 149: 863–873. 19074625 10.1104/pp.108.131516 PMC2633839

[42] Kapulnik Y, Delaux PM, Resnick N, Mayzlish-Gati E, Wininger S, et al (2011) Strigolactones affect lateral root formation and root-hair elongation in Arabidopsis. Planta 233: 209–216. 21080198 10.1007/s00425-010-1310-y

[43] Carol RJ, Dolan L (2002) Building a hair: tip growth in Arabidopsis thaliana root hairs. Philos Trans R Soc Lond B Biol Sci 357: 815–821. 12079677 10.1098/rstb.2002.1092 PMC1692992

[44] Ruyter-Spira C, Kohlen W, Charnikhova T, van Zeijl A, van Bezouwen L, et al (2011) Physiological effects of the synthetic strigolactone analog GR24 on root system architecture in Arabidopsis: another belowground role for strigolactones? Plant physiology 155: 721–734. 21119044 10.1104/pp.110.166645 PMC3032462

[45] Rasmussen A, Depuydt S, Goormachtig S, Geelen D (2013) Strigolactones fine-tune the root system. Planta 238: 615–626. 23801297 10.1007/s00425-013-1911-3

[46] Hu Z, Yan H, Yang J, Yamaguchi S, Maekawa M, et al (2010) Strigolactones Negatively Regulate Mesocotyl Elongation in Rice during Germination and Growth in Darkness Plant Cell Physiol. 51: 1136–1142. 10.1093/pcp/pcq075 PMC2900821 20498118

[47] Arite T, Kameoka H, Kyozuka J (2012) Strigolactone Positively Controls Crown Root Elongation in Rice. Journal of Plant Growth Regulation 31: 165–172.

[48] Zwanenburg B, Nayak SK, Charnikhova TV, Bouwmeester HJ (2013) New strigolactone mimics: Structure-activity relationship and mode of action as germinating stimulants for parasitic weeds. Bioorg Med Chem Lett 23: 5182–5186. 23920440 10.1016/j.bmcl.2013.07.004

[49] Booker J, Sieberer T, Wright W, Williamson L, Willett B, et al (2005) MAX1 encodes a cytochrome P450 family member that acts downstream of MAX3/4 to produce a carotenoid-derived branch-inhibiting hormone. Developmental cell 8: 443–449. 15737939 10.1016/j.devcel.2005.01.009

[50] Nelson DC, Scaffidi A, Dun EA, Waters MT, Flematti GR, et al (2011) F-box protein MAX2 has dual roles in karrikin and strigolactone signaling in Arabidopsis thaliana. Proceedings of the National Academy of Sciences of the United States of America 108: 8897–8902. 21555559 10.1073/pnas.1100987108 PMC3102411

[51] Waters MT, Smith SM, Nelson DC (2011) Smoke signals and seed dormancy: where next for MAX2? Plant signaling & behavior 6: 1418–1422. 22019642 10.4161/psb.6.9.17303 PMC3258081

[52] Prandi C, Rosso H, Lace B, Occhiato EG, Oppedisano A, et al (2013) Strigolactone analogs as molecular probes in chasing the (SLs) receptor/s: design and synthesis of fluorescent labeled molecules. Molecular plant 6: 113–127. 23180673 10.1093/mp/sss133

[53] Ashton NW, Cove DJ (1977) The isolation and preliminary characterisation of auxotrophic and analogue resistant mutants of the moss, Physcomitrella patens. Molecular General Genetics 154: 87–95.

[54] Ashton NW, Grimsley NH, Cove DJ (1979) Analysis of gametophytic development in the moss, Physcomitrella patens, using auxin and cytokinin resistant mutants. Planta 144: 427–435. 24407386 10.1007/BF00380118

[55] Chen VX, Boyer FD, Rameau C, Retailleau P, Vors JP, et al (2010) Stereochemistry, total synthesis, and biological evaluation of the new plant hormone solanacol. Chemistry 16: 13941–13945. 21108265 10.1002/chem.201002817

[56] Fox J (2005) The R commander: A basic-statistics graphical user interface to R. Journal of Statistical Software. 14: 1–42.

