# Supplementary figures and images for: Strigolactones Inhibit Caulonema Elongation and Cell Division in the Moss Physcomitrella patens
Source: PLoS One. 2014 Jun 9;9(6):e99206. doi: 10.1371/journal.pone.0099206 (PMC4049778; doi:10.1371/journal.pone.0099206)

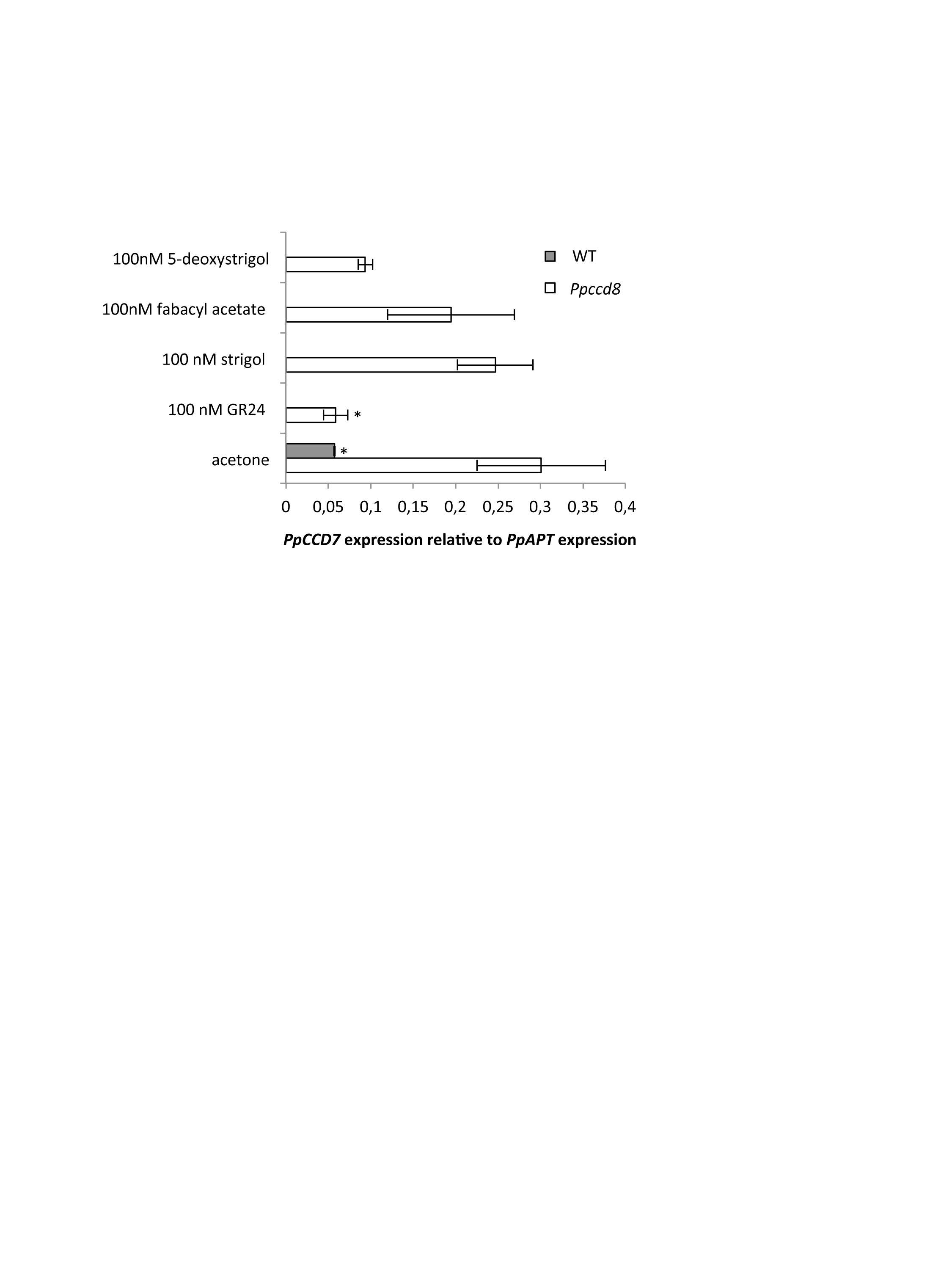

Supplement: Figure S1 — Effect of various strigolactones and analogues on relative PpCCD7 expression 2 h after application of 100 nM SL in WT and SL-deficient ( Ppccd8 ) mutant plants in the light. Data are means ± SE (n = 3 biological replicates). Asterisks denote significant differences from Ppccd8 treated with acetone (* P<0.05, one-way ANOVA). (TIF) [file pone.0099206.s001.tif]

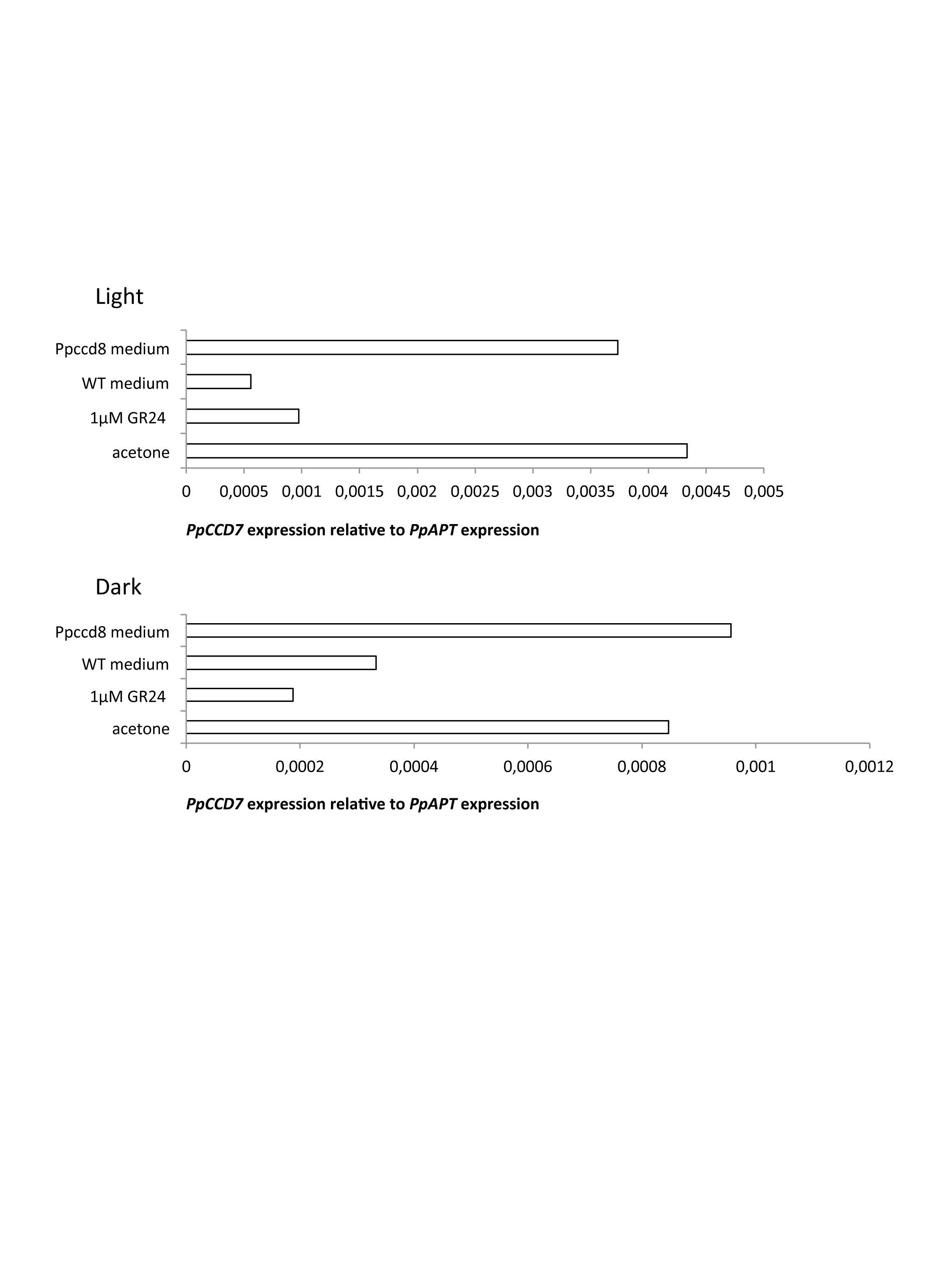

Supplement: Figure S2 — Relative PpCCD7 expression of Ppccd8 mutant grown in the light (top) or in the dark (bottom), 6 h after addition of 1 µM GR24 (control: acetone) or following transfer to medium on which WT or Ppccd8 mutant plants (fragmented protonema using 7-day-old culture) had grown for 20 days. Plants from three Petri dishes were used for each condition. A biological replication of the experiment gave similar results. (TIF) [file pone.0099206.s002.tif]
